# Early prediction models for prognosis of diabetic ketoacidosis in the emergency department: A protocol for systematic review and meta-analysis

**DOI:** 10.1097/MD.0000000000026113

**Published:** 2021-05-28

**Authors:** Qin Li, Lin Lv, Yao Chen, Yiwu Zhou

**Affiliations:** a Department of Emergency Medicine, Emergency Medical Laboratory; b School of Nursing, West China Hospital, Sichuan University, Chengdu, Sichuan, China.

**Keywords:** diabetic ketoacidosis protocol, early, emergency, meta-analysis, prediction models, prognosis, systematic review

## Abstract

**Background:**

Diabetic ketoacidosis (DKA) is one of the most serious complications after diabetes poor control, which seriously threatens human life, health, and safety. DKA can rapidly develop within hours or days leading to death. Early evaluation of the prognosis of DKA patients and timely and effective intervention are very important to improve the prognosis of patients. The combination of several variables or characteristics is used to predict the poor prognosis of DKA, which can allocate resources reasonably, which is beneficial to the early classification intervention and clinical treatment of the patients.

**Methods:**

For the acquisition of required data of eligible prospective/retrospective cohort study or randomized controlled trials (RCTs), we will search for publications from PubMed, Web of science, EMBASE, Cochrane Library, Google scholar, China national knowledge infrastructure (CNKI), Wanfang and China Science and Technology Journal Database (VIP). Two independent reviewers will read the full English text of the articles, screened and selected carefully, removing duplication. Then we evaluate the quality and analyses data by Review Manager (V.5.4). Results data will be pooled and meta-analysis will be conducted if there's 2 eligible studies considered.

**Results:**

This systematic review and meta-analysis will evaluate the value of the prediction models for the prognosis of DKA in the emergency department.

**Conclusions:**

This systematic review and meta-analysis will provide clinical basis for predicting the prognosis of DKA. It helps us to understand the value of predictive models in evaluating the early prognosis of DKA. The conclusions drawn from this study may be beneficial to patients, clinicians, and health-related policy makers.

**Study registration number:**

INPLASY202150023.

## 1 Introduction

Diabetic ketoacidosis (DKA) is one of the most serious and life-threatening hyperglycemia emergencies in diabetes.^[1–3]^ Both type 1 and type 2 diabetes can occur DKA, but are more common in young people with type 1 diabetes.^[4,5]^ A report from the American Youth Diabetes Study (SEARCH for Diabetes In Youth study) shows: diabetic patients under 20 years of age are at a high risk of DKA (about 29% for type 1 and 10% for type 2).^[6]^ Among Scottish adults under the age of 50, DKA was identified as the single factor leading to death (21.7% for women and 29.4% for men).^[7]^ The mortality rate of single DKA attack is about 5.2%, and that DKA recurrent attack is about 23.4%.^[8,9]^ There are 3 main diagnostic bases for DKA: first, blood glucose level; second, ketone body (including urine ketone body and serum ketone body); third, acid poisoning.^[10,11]^ The patient with DKA can progress quickly within few hours, and even some patients have developed to critical state without being aware of it. The clinical symptoms of DKA are diverse, mild condition can be manifested as dry mouth drink, polyuria, nausea, vomiting, abdominal pain, fatigue and other single symptoms or multiple symptoms, severe illness can be accompanied by changes in conscious state and shock and other multiple organ function injury.^[6,12–15]^ Early evaluation of DKA prognosis is important.^[16–18]^ A variable or feature used to assess the severity of DKA can help medical workers allocate medical resources reasonably and intervene early in the treatment of DKA.^[19–23]^ But the combination of several variables or characteristics has a better evaluation of the prediction effect.^[11,16]^

Up to now, there is no systematic review or meta-analysis of prognostic prediction models of DKA. We aim to systematically review and critically evaluate existing predictive models of DKA, especially the prognostic models of the disease.

## 2 Methods

### 2.1 Study registration

This systematic review was recorded in the international platform of registered systematic review and meta-analysis protocols (INPLASY) on May 6, 2021 with the registration number of INPLASY202150023 (doi:10.37766/inplasy2021.5.0023). This protocol was drafted and reported in accordance with the PRISMA protocols 2015 statement which is represented for Preferred Reporting Items for Systematic Review and Meta-Analyses.^[24]^

### 2.2 Eligibility criteria for study selection

We will collect all cross-sectional studies and clinic studies that predict or analyzed the admission and death of patients with DKA. Children or pregnant women donot meet the inclusion criteria. No restrictions will be placed on sex or gender, race, comorbidities, or other characteristics. Animal studies, case reports, cadaver studies, letters, comments, protocols, guidelines, unpublished articles, and review papers will be excluded. This search will be limited to reports in English, and for which full-text access is available. Participants who are included in the articles we selected should be diagnosed with DKA. This study will follow the PICO strategy that considering the eligibility criteria adopted in the population of the study, Intervention, Comparison, Result, and Study Design according to the details (Table 1).

**Table 1:** The PICOS description about study.

PICOS description		
PICOS	Abbreviation	Elements
Patient population	P	Adult
Intervention/exposure	I	Patients with diabetic ketoacidosis
Comparison/control	C	Patients without diabetic ketoacidosis
Outcome	O	Risk of disease due to diabetic ketoacidosis
Study design	S	Prospective/retrospective cohort study, RCTs

### 2.3 Search strategy

At first, the collection of bibliographic data will be made in the electronic databases: Web of science, PubMed, EMBASE, Cochrane Library, Google scholar, CNKI, Wanfang, and VIP. We use the available publications of the DKA living systematic review for a list of keywords. The words are considered: DKA, diagnostic, prognostic, prediction, prediction model, regression, score, artificial intelligence, algorithm, deep learning, machine learning. We make the search terms by combining the words above:

#1 diabetic ketoacidosis
#2 diagnostic OR imaging OR prognostic OR prognosis OR prediction OR prediction model OR mortality OR regression OR score OR artificial intelligence OR algorithm OR deep learning OR machine learning
#3 APACH II OR PSI ORSOFA OR qSOFA OR SAPS
#4 english NOT animal NOT meternal
#1 AND #2 AND #3 AND #4

### 2.4 Selection of studies

**Figure 1:**
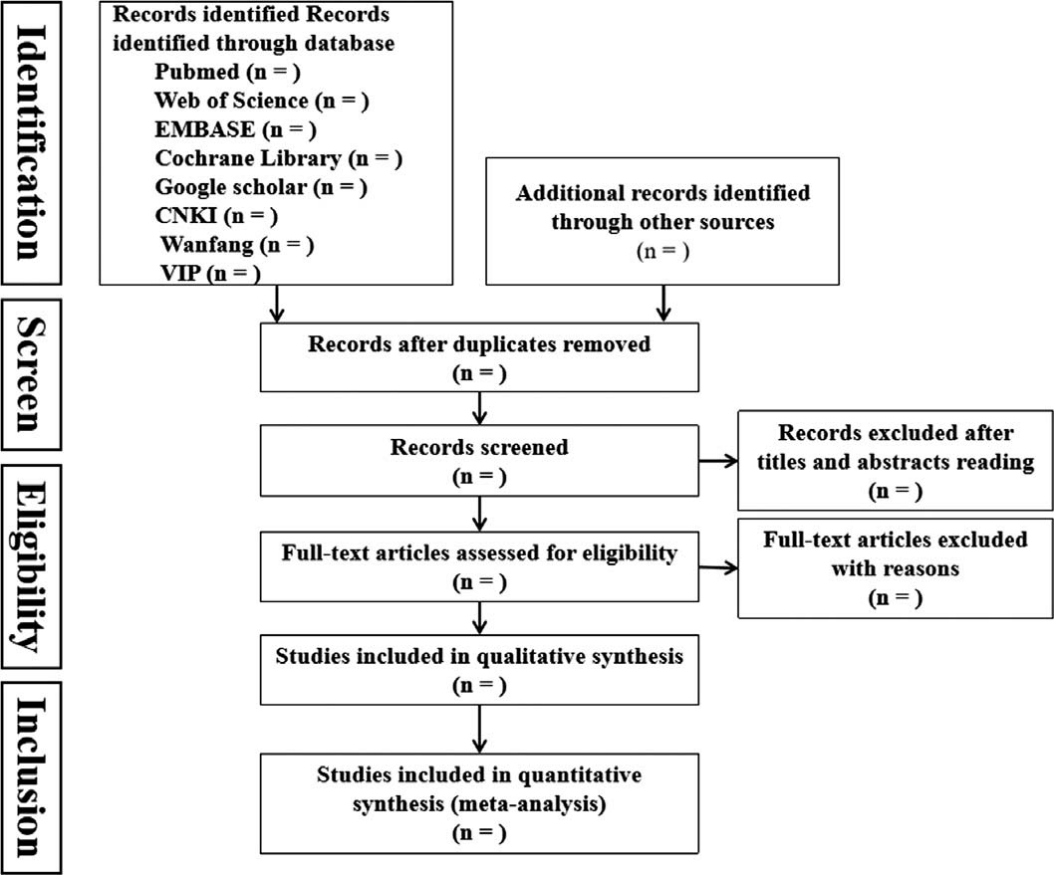
Flow diagram of study selection process.

The preliminary documents are obtained by looking through the titles and abstract, removing the duplications. For the further screening, the 2 reviewers will read the full text of the articles which are selected carefully, removing the unsatisfied articles and sending an email to ask author for the full text or the details. Any disagreements will be arbitrated by a third reviewer. The whole process of study selection is presented in the flow chart following the a PRISMA principle (Fig. 1).

### 2.5 Data extraction and management

Two independent authors will extract the following descriptive raw information from the selected studies, including author information, study area, study time, study type, study design, setting of study, sample size, participant characteristics, primary and secondary outcomes (needing for mechanical ventilation, needing for ICU care, or dead), area under curve (AUC). Another researcher will solve the divergence between the first 2 reviewers. If necessary, we will abandon the extraction of incomplete data.

### 2.6 Assessment of risk of bias

In order to achieve a consistency (at least 80%) of risk of bias assessment, the risk of bias assessors will pre-evaluate a sample of qualified studies. Results of the pilot risk of bias will be discussed among review authors and assessors. The Grading of Recommendations, Assessment, Development and Evaluation (GRADE) assessment tool will be used for conducting an appraisal of the studies’ methodological quality. Every selected study will be evaluated by 2 reviewers independently, a third one as a consulter. The GRADE evaluation system included bias risk; heterogeneity; indirectness; imprecision; publication bias. And each level of evidence is divided into “very low,” “low,” “moderate,” or “high” judgment.

### 2.7 Data synthesis and analysis

For qualified articles, we would like to merge the collected data according to characteristics of eligible trials. In line with the Cochrane guideline, we will express risk ratio with 95% confidence intervals (95% CI) using fixed effect model. Besides the random effect model will be used for continuous outcomes because of clinical heterogeneity. Statistical heterogeneity will be investigated using Chi-squared test and *I*^2^ statistic (<25%, no heterogeneity; 25%–50%, moderate heterogeneity; and >50%, strong heterogeneity). We will assess possible publication bias using the Egger funnel plot. All data will be performed by using Review Manager ((RevMan version 5.4.0, The Cochrane Collaboration, 2020) software and *P* value < .05 will be considered statistically significant.

### 2.8 Ethics and dissemination

Because the study is a secondary analysis, it will not involve patient and public information, and hence does not require ethical review. Ethical approval is unnecessary because this is a literature-based study.

## 3 Discussion

At home and abroad, DKA is one of the most life-threatening acute diseases in patients with poor blood glucose control. DKA occurs regardless of age and sex, it is due to a relative or absolute deficiency of insulin, as well as excessive hormonal regulation, leading to varying degrees of hypertonic dehydration, disorders of sugar-salt electrolytes, and intracellular protein breakdown,^[6,25]^ and then different clinical manifestations. There are some studies on the evaluation of the treatment of DKA.^[6,11,26,27]^ However, there is no systematic review or meta-analysis to the prognostic prediction model of DKA. Five English databases and 3 Chinese databases will be searched to avoid missing any potential eligible studies. Therefore, we carried out this systematic review to help clinicians to reasonably allocate medical resources by predicting different prognosis of DKA and to provide clinical evidence for timely and effective treatment.

## Author contributions

**Conceptualization:** Qin Li, Lin Lv, Yiwu Zhou, Yao Chen.

**Data curation:** Qin Li, Lin Lv.

**Formal analysis:** Qin Li, Lin Lv, Yiwu Zhou.

**Funding acquisition:** Qin Li.

**Investigation:** Qin Li, Lin Lv, Yao Chen.

**Methodology:** Qin Li, Yao Chen.

**Project administration:** Qin Li, Yao Chen.

**Resources:** Qin Li, Lin Lv.

**Software:** Yiwu Zhou, Lin Lv.

**Supervision:** Qin Li, Yiwu Zhou.

**Validation:** Yiwu Zhou, Lin Lv.

**Visualization:** Qin Li, Yao Chen.

**Writing – original draft:** Qin Li, Lin Lv.

**Writing – review & editing:** Qin Li, Yiwu Zhou, Yao Chen.
